# Supplementary material for: Machine learning sequence prioritization for cell type-specific enhancer design
Source: eLife. 2022 May 16;11:e69571. doi: 10.7554/eLife.69571 (PMC9110026; doi:10.7554/eLife.69571)
Supplement: Transparent reporting form [file elife-69571-transrepform1.pdf]

# *eLife's* transparent reporting form

We encourage authors to provide detailed information *within their submission* to facilitate the interpretation and replication of experiments. Authors can upload supporting documentation to indicate the use of appropriate reporting guidelines for health-related research (see EQUATOR Network), life science research (see the BioSharing Information Resource), or the ARRIVE guidelines for reporting work involving animal research. Where applicable, authors should refer to any relevant reporting standards documents in this form.

If you have any questions, please consult our Journal Policies and/or contact us: editorial@elifesciences.org.

**Sample-size estimation**

- You should state whether an appropriate sample size was computed when the study was being designed
- You should state the statistical method of sample size computation and any required assumptions
- If no explicit power analysis was used, you should describe how you decided what sample (replicate) size (number) to use

Please outline where this information can be found within the submission (e.g., sections or figure legends), or explain why this information doesn't apply to your submission:

We followed the current ENCODE standards for ATAC-seq, which are described in the Materials and Methods section "Experimental design".

**Replicates**

- You should report how often each experiment was performed
- You should include a definition of biological versus technical replication
- The data obtained should be provided and sufficient information should be provided to indicate the number of independent biological and/or technical replicates
- If you encountered any outliers, you should describe how these were handled
- Criteria for exclusion/inclusion of data should be clearly stated
- High-throughput sequence data should be uploaded before submission, with a private link for reviewers provided (these are available from both GEO and ArrayExpress)

Please outline where this information can be found within the submission (e.g., sections or figure legends), or explain why this information doesn't apply to your submission:

Replicate information for the ATAC-seq data is described in the Materials and Methods section under "Experimental design" and further details are available in Supplementary File 1. The ATAC-seq data is available on GEO, accession GSE171549, reviewer token = sdalwawczhcnrut. For image quantification data, replicate information is described in the Figure 2 legend and more detailed information can be found in Figure 2- Source Data 2.

**Statistical reporting**

- Statistical analysis methods should be described and justified
- Raw data should be presented in figures whenever informative to do so (typically when N per group is less than 10)
- For each experiment, you should identify the statistical tests used, exact values of N, definitions of center, methods of multiple test correction, and dispersion and precision measures (e.g., mean, median, SD, SEM, confidence intervals; and, for the major substantive results, a measure of effect size (e.g., Pearson's r, Cohen's d)
- Report exact p-values wherever possible alongside the summary statistics and 95% confidence intervals. These should be reported for all key questions and not only when the p-value is less than 0.05.

Please outline where this information can be found within the submission (e.g., sections or figure legends), or explain why this information doesn't apply to your submission:

Image quantification statistics (Figure 2d-e, Figure 6d) and Ns are described in the Figure legends and further detailed in Figure 2- Source Data 2. We describe the statistical methods used for differential chromatin accessibility from ATAC-seq data in Materials & Methods sections "ATAC-seq data processing" and "snATAC-seq data processing" and lists of resulting p-values are available in Figure 3- Source Data 1, Figure 4- Source Data 1, Figure 4- Source Data 4, and Figure 4- Source Data 5. Motif associations and motif matches with position weight matrices are described in the Materials and Methods section "Model interpretation" and the related source data are available in Figure 5- Source Data 1 and Figure 5- Source Data 2. For comparisons between SNAIL, cSNAIL ATAC-seq, and snATAC-seq data (Figure 3c-d, Figure 4e,h, Figure 4- figure supplement 2), the statistical tests and confidence intervals are mentioned in the figure legends and the related methods are in the Materials and Methods section "Evaluation of SC1 and SC2 ATAC-seq".

(For large datasets, or papers with a very large number of statistical tests, you may upload a single table file with tests, Ns, etc., with reference to sections in the manuscript.)

**Group allocation**

- Indicate how samples were allocated into experimental groups (in the case of clinical studies, please specify allocation to treatment method); if randomization was used, please also state if restricted randomization was applied
- Indicate if masking was used during group allocation, data collection and/or data analysis

Please outline where this information can be found within the submission (e.g., sections or figure legends), or explain why this information doesn't apply to your submission:

All animals in these experiments were healthy individuals from the same colony. We describe experimental group segmentation in Supplementary File 1.

**Additional data files ("source data")**

- We encourage you to upload relevant additional data files, such as numerical data that are represented as a graph in a figure, or as a summary table
- Where provided, these should be in the most useful format, and they can be uploaded as "Source data" files linked to a main figure or table
- Include model definition files including the full list of parameters used
- Include code used for data analysis (e.g., R, MatLab)
- Avoid stating that data files are "available upon request"

Please indicate the figures or tables for which source data files have been provided:

Source data related to Figures 1-5 have been provided in the form of excel files. They include model definitions (Figure 1- Source Data 1 and Figure 1- Source Data 2) and numerical data represented in main figures (Figure 1- Source Data 3, Figure 1- Source Data 4, Figure 2- Source Data 1, Figure 2- Source Data 2, Figure 3- Source Data 1, Figure 4- Source Data 1, Figure 4- Source Data 2, Figure 4- Source Data 3, Figure 4- Source Data 4, Figure 4- Source Data 5, Figure 5- Source Data 1, Figure 5- Source Data 2, and Figure 5- Source Data 3).
